# LC-MS/MS Method for Simultaneous Quantification of Dexmedetomidine, Dezocine, and Midazolam in Rat Plasma and Its Application to Their Pharmacokinetic Study

**DOI:** 10.1155/2018/3184759

**Published:** 2018-05-20

**Authors:** Wenjuan Cui, Qin Liu, Shan Xiong, Lujun Qiao

**Affiliations:** ^1^Intensive Care Unit, Shengli Oilfield Central Hospital, Dongying, Shandong 257034, China; ^2^Institute of Materia Medica, Shandong Academy of Medical Sciences, Jinan, Shandong 250062, China; ^3^Key Laboratory for Biotech-Drugs Ministry of Health, Jinan, Shandong 250062, China; ^4^Key Laboratory for Rare and Uncommon Diseases of Shandong Province, Jinan, Shandong 250062, China

## Abstract

A simple, sensitive, and accurate LC-MS/MS method was established and validated for the simultaneous quantification of dexmedetomidine, dezocine, and midazolam in rat plasma. Chromatographic separation was achieved on a C18 column (50 mm × 2.1 mm, 3 *µ*m) using a mobile phase composed of water (containing 0.1% formic acid) and acetonitrile. The lower limits of quantification were 0.1, 0.1, and 0.2 ng/mL for dexmedetomidine, dezocine, and midazolam in rat plasma, respectively. The analytes were determined with selected reaction monitoring under positive ionization mode. The intra- and interday precision and accuracy were all within acceptable limits during the entire validation, and the stability of analytes was acceptable under various storage conditions. The validated method was successfully applied in pharmacokinetic studies of dexmedetomidine, dezocine, and midazolam following intravenous injection.

## 1. Introduction

In intensive care unit (ICU), it is becoming increasingly important to measure and manage delirium, pain, and agitation in adult patients. Actually, guidelines for the treatment of delirium, pain, and agitation in critically ill patients do not provide recommendations on pharmacologic treatment [1, 2]. For most patients in ICU, a safer and more effective strategy that ensures patients ease while maintaining a light level of sedation is related to the improvement of clinical outcomes [3]. In many Chinese hospitals, the combined application of dexmedetomidine, dezocine, and midazolam is used for the treatment of delirium, pain, and agitation in adult patients in ICU, and the treatment effect shows these agents have made significant advances in critically ill patients [4–6].

But up to now, being lack of enough attention and over emphasis on efficacy, the pharmacokinetic characteristics of dexmedetomidine, dezocine, and midazolam combination in ICU patients were still unclear. In the previous studies, they had been reported for the pharmacokinetic study of dexmedetomidine [7], dezocine [8], and midazolam [9] in the patients and healthy volunteers, respectively. There had been only one study done to research the plasma concentrations and sedative effects of a dexmedetomidine, midazolam, and butorphanol combination after transnasal administration in healthy rabbits [10].

The objective of the present study was to establish a simple, sensitive, and accurate LC-MS/MS method for simultaneous determination of dexmedetomidine, dezocine, and midazolam in rat plasma and investigate the pharmacokinetic characteristics of dexmedetomidine, dezocine, and midazolam in rats. Then, the method would lay the foundations for monitoring of dexmedetomidine, dezocine, and midazolam in patient plasma in ICU.

## 2. Materials and Methods

### 2.1. Materials

Dexmedetomidine and midazolam were obtained from Sigma-Aldrich Chemical Company (St. Louis, MO, USA). Dezocine and carteolol (IS) were purchased from the National Institute for the Control of Pharmaceutical and Biological Products (Beijing, China). HPLC-grade methanol and acetonitrile were obtained from Tedia (Fairfield, OH, USA). HPLC-grade formic acid was purchased from Kermel Chemical Reagent Co., Ltd. (Tianjin, China). All other chemicals were of analytic grade or better.

### 2.2. Instruments and Analytical Conditions

**Figure 1:**
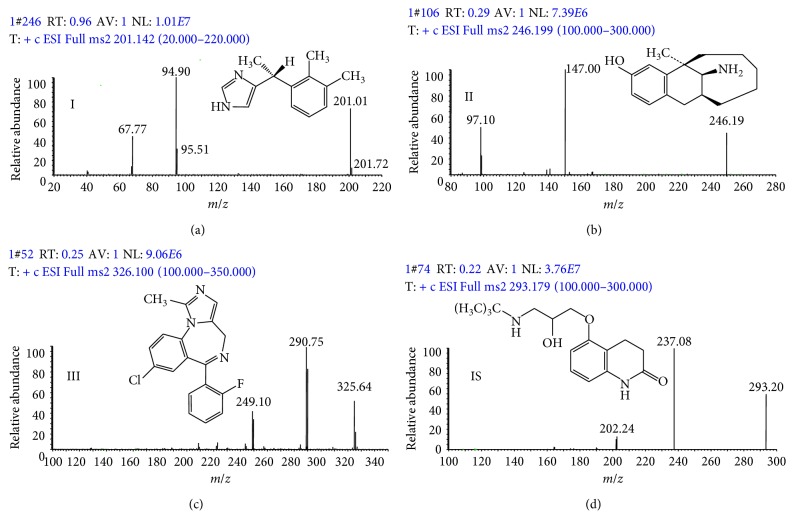
Chemical structures and positive-ion electrospray mass spectra of dexmedetomidine (I), dezocine (II), midazolam (III), and IS.

A TSQ Quantum Ultra mass spectrometric detector with electrospray ionization (ESI) interface (Thermo Scientific, USA) was coupled with a Dionex Ultimate 3000 ultra-performance liquid chromatography system consisting of an LPG-3400SDN pump, a WPS-3000TSL autosampler, and a TCC-3000RS column compartment. The separation of analytes and IS was achieved by using a Hypersil GOLD C18 column (50 mm × 2.1 mm, 3 *µ*m; Thermo Scientific, USA) that was maintained at 25°C. The water (containing 0.1% formic acid and solvent A) and acetonitrile (solvent B) was used as mobile phase with gradient elution. The gradient was as follows: 0 min 5% B, 0.5 min 5% B, 2.0 min 80% B, 2.1 min 5% B, and 3.0 min 5% B, and then stopped. The flow rate was set at 0.4 mL/min. The select reaction monitoring (SRM) conditions (source parameters) were defined as follows: spray voltage 3.5 kV, vaporizer temperature 250°C, and capillary temperature 300°C. The optimized mass spectrometric parameters and mass spectra for all the analytes are shown in Table 1 and Figure 1, respectively. All the operations, acquiring and analysis of data, were controlled using Xcalibur software (Thermo Scientific, USA).

### 2.3. Preparation of Standard Solutions, Calibration Samples, and Quality Control Samples

Standard mixed stock solutions of dexmedetomidine, dezocine, and midazolam were prepared in DMSO at 1.0, 1.0, and 2.0 mg/mL, respectively, and then serially diluted with methanol to obtain the working solutions. The carteolol (IS) stock solution of 20 mg/mL was prepared in double-distilled water and further diluted with methanol to 1.0 *µ*g/mL. All solutions were kept at 4°C before use. The diluted solutions were mixed with blank rat plasma (1 : 99) at final concentrations of 0.1, 0.5, 1.0, 10.0, 20.0, and 100 ng/mL for dexmedetomidine; 0.1, 0.5, 1.0, 10.0, 20.0, and 100 ng/mL for dezocine; and 0.2, 1.0, 2.0, 20.0, 40.0, and 200 ng/mL for midazolam. The quality control (QC) samples were prepared in blank rat plasma at four different concentration levels, lower limit of quantification (LLOQ, 0.1/0.1/0.2 ng/mL), low QC (0.2/0.2/0.4 ng/mL), medium QC (5.0/5.0/10.0 ng/mL), and high QC (80.0/80.0/160 ng/mL), for dexmedetomidine, dezocine, and midazolam, respectively.

### 2.4. Preparation of Plasma Samples

Frozen plasma samples were thawed at room temperature and vortexed for 1 min. 100 *μ*L of rat plasma was mixed with 10 *μ*L of IS solution (1.0 *μ*g/mL) and 550 *μ*L of ethyl acetate. Then, the extraction was carried out by vortex mixing for 3 min and centrifuging at 14,000 rpm for 10 min. The organic phases were transferred into clean polythene tubes and evaporated to dryness with a gentle nitrogen stream at 40°C. The residues were reconstituted in 100 *μ*L of methanol followed by vortex mixing. After centrifuging at 14,000 rpm for 10 min at 4°C, 10 *μ*L of the supernatant was injected onto the LC-MS/MS system for analysis.

### 2.5. Method Validation

A full method validation of the assay for the analyte determination in rat plasma was performed according to the China Food and Drug Administration guidelines for the preclinical pharmacokinetic study with respect to selectivity, linearity, precision, accuracy, recovery, matrix effect, stability, and dilution integrity [11].

### 2.6. Application of the Method

Six male Sprague-Dawley rats weighing 180–220 g were supplied by the Shandong Laboratory Animal Center of Shandong Academy of Medical Sciences (certificate number SCXK (Shandong) 2014-0007). Rats were housed in environmentally controlled quarters (about 23°C–25°C) under a 12 h: 12 h light-dark cycle for at least 1 week before experiment. All rats were fasted but with freely available water for 12 h prior to pharmacokinetic study. The animal welfare and experimental procedures were approved by the Animal Ethics Committee at Institute of Materia Medica, Shandong Academy of Medical Sciences (Jinan, China).

The established LC-MS/MS method was applied for the plasma concentration determination of dexmedetomidine (20 *μ*g/kg), dezocine (80 *μ*g/kg), and midazolam (400 *μ*g/kg) after simultaneous intravenous administration through tail vein to healthy SD rats (*n*=6). Blood samples were drawn into heparinized tubes before and at 2, 5, 15, 30, 45, 60, 120, 180, 240, 360, and 480 min after a single intravenous administration. The samples were immediately centrifuged at 3500 rpm for 10 min at 4°C. The plasma was separated and stored below −20°C until LC-MS/MS analysis [12, 13].

## 3. Results and Discussion

### 3.1. Optimization of LC-MS Conditions

After lots of preliminary experiments, a Hypersil GOLD C18 column (50 mm × 2.1 mm, 3 *µ*m) was chosen to achieve satisfactory chromatographic results. The mobile phase consisted of water (containing 0.1% formic acid) and acetonitrile with gradient elution, which played a great role in achieving good peak symmetry with appropriate retention time and high MS sensitivity.

In addition, owing to the complex nature of plasma, the pretreatment of plasma sample is a significant step to remove protein and potential interferences prior to LC-MS/MS analysis. The extraction conditions, including protein precipitation and liquid-liquid extraction, were investigated. Although the extraction procedure of protein precipitation required less time and could decrease the cost of the assay, the sensitivity and specificity could not meet the requirements of the research. For liquid-liquid extraction, the ratio of ethyl acetate to plasma volume was investigated. Finally, ethyl acetate at the ratio of 1 : 5.5 to plasma volume proved to be the best extraction method, as it yielded high recovery, negligible matrix effect for the analytes, and acceptable evaporation time. Additionally, 3 min was proved to be the most appropriate time for vortex mixing. As a result, the liquid-liquid extraction showed the advantages of specificity, sensitivity, stability, and accuracy.

### 3.2. Method Validation

#### 3.2.1. Specificity and Selectivity

**Figure 2:**
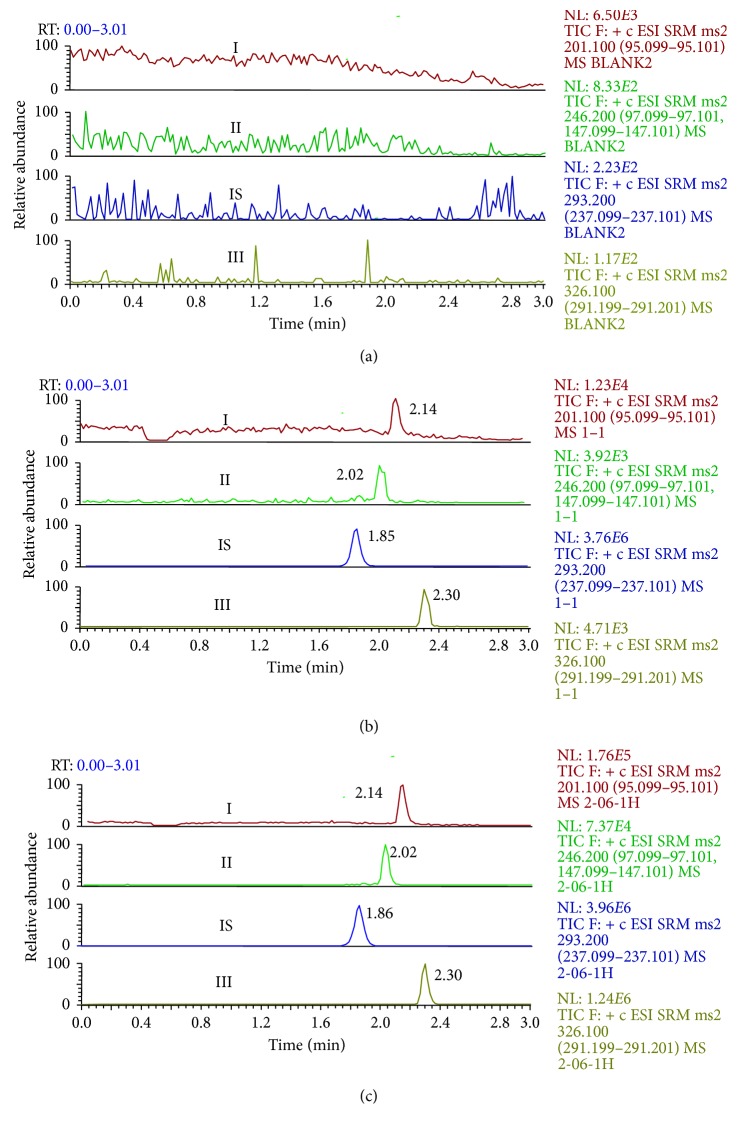
SRM chromatograms of dexmedetomidine (I), dezocine (II), midazolam (III), and IS in rat plasma. (a) Chromatographic profile of blank rat plasma; (b) chromatographic profile of rat plasma spiked with dexmedetomidine (I, 0.1 ng/mL), dezocine (II, 0.1 ng/mL), midazolam (III, 0.2 ng/mL), and IS; (c) chromatographic profile of a plasma sample 1 h after administration of the analytes to a rat.

The retention time of dexmedetomidine, dezocine, midazolam, and carteolol (IS) were 2.14, 2.02, 2.30, and 1.86 min, respectively, and all samples were found to have no interferences from endogenous substances at the retention time of the analytes. The SRM chromatograms of the three analytes and IS are shown in Figure 2.

#### 3.2.2. Linearity and LLOQs

To evaluate linearity, the calibration curves for each analyte were prepared and assayed from the ratio of the analyte peak area to the IS peak area (*y*) versus concentration (*x*). The LLOQ was determined as the lowest concentration with a signal-to-noise (S/N) ratio of 10, and the accuracy was between 80.00 and 120.00% by analyzing six replicates. The linear ranges, correlation coefficients, weight factors, regression equations, and LLOQs for all analytes are summarized in Table 2.

#### 3.2.3. Precision and Accuracy

The QC samples at four concentration levels (lower limit of quantitation (LLOQ), low, medium, and high) were selected and analyzed to evaluate the intraday and interday precision and accuracy on three consecutive days (*n*=6), which could cover the whole range of the calibration graph. For dexmedetomidine, dezocine, and midazolam, the intraday and interday accuracy was between 92.60 and 119.00%, and the intraday and interday RSD was <14.28% (Table 3).

#### 3.2.4. Extraction Recovery and Matrix Effect

Extraction recovery was assessed by comparing the MS responses from QC samples at three concentration levels (low, medium, and high) with the responses of dexmedetomidine, dezocine, and midazolam from the samples spiked after extraction at the same level. The matrix effect was measured via comparison of MS responses from the samples spiked after extraction with the responses from neat samples at the same concentration. The results showed that they were all within the acceptable limit for extraction recovery, and there was no significant matrix effect on dexmedetomidine, dezocine, and midazolam in this method (Table 4).

#### 3.2.5. Stability and Dilution Integrity

The stability of analytes in rat plasma was evaluated at the following temperature and timing conditions: 6 h at ambient temperature, 10 d at −20°C, three freeze-thaw cycles, and 24 h at 4°C in autosampler. The measured concentrations (QC samples) of dexmedetomidine, dezocine, and midazolam were all within acceptable limits during the entire validation (Table 5).

Six replicate samples, with the concentrations of 500, 500, and 1000 ng/mL for dexmedetomidine, dezocine, and midazolam, respectively, were diluted 10-fold with blank rat plasma. The diluted samples were analyzed and the estimated accuracy and precision were compared to the theoretical value. The precision (RSD) was less than 15%, and the accuracy was within 85–115% for all the analytes.

### 3.3. Pharmacokinetic Studies

**Figure 3:**
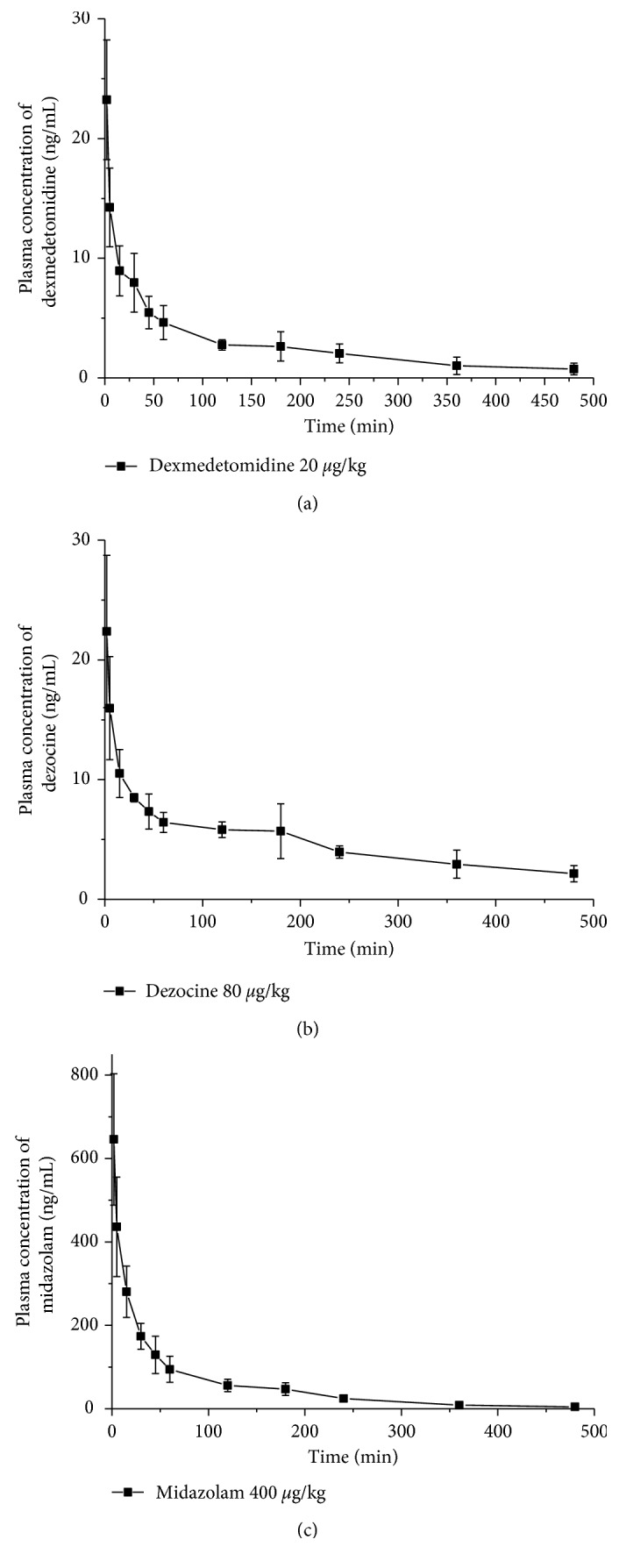
Plasma concentration-time profiles of dexmedetomidine, dezocine, and midazolam following single intravenous administration in SD rats (*n*=6).

The plasma concentration-time profiles of dexmedetomidine, dezocine, and midazolam following single intravenous administration in SD rats are presented in Figure 3. The main pharmacokinetic parameters of dexmedetomidine, dezocine, and midazolam were processed by a noncompartmental model using the DAS 2.0 software package (Mathematical Pharmacology Professional Committee of China, Shanghai, China). The pharmacokinetic parameters including AUC_(0–8 h)_, MRT_(0–8 h)_, *T*_1/2z_, *C*_2 min_, *V*_d_, and *C*_Lz/F_ are summarized in Table 6. All results were expressed as arithmetic mean ± standard deviation (SD).

## 4. Conclusion

In the present study, a simple, sensitive, and accurate LC-MS/MS method for the simultaneous quantification of dexmedetomidine, dezocine, and midazolam in rat plasma has been developed and validated, and then the LC-MS/MS assay was successfully applied to the pharmacokinetic studies of dexmedetomidine, dezocine, and midazolam in rats after combination administration. A simple liquid-liquid extraction process was used, and the total run time was no more than 3.0 min. The results were of great importance and valuable reference due to providing preliminary nonclinical evidence from animals after a single dosing. The method was probably used to determine the concentration of dexmedetomidine, dezocine, and midazolam in patient plasma in ICU.

## Figures and Tables

**Table 1:** The optimized mass spectrometric parameters for dexmedetomidine, dezocine, midazolam, and IS.

Analytes	Parent ion (*m*/*z*)	Product ion (*m*/*z*)	Collision energy (V)
Dexmedetomidine	201.01	94.90	17
Dezocine	246.19	147.00	18
Midazolam	325.64	290.75	25
IS	293.20	237.08	13

**Table 2:** Linear ranges, correlation coefficients, weight factors, regression equations, and LLOQs for all analytes in rat plasma.

Analytes	Linear ranges (ng/mL)	*r* ^2^	Weight factors	Regression equations	LLOQs (ng/mL)
Dexmedetomidine	0.1–100	0.9975	1/*X*^2^	*Y* = 4.52 × 10^−5^ + 4.99 × 10^−3^*X*	0.1
Dezocine	0.1–100	0.9962	1/*X*^2^	*Y* = 2.33 × 10^−5^ + 1.19 × 10^−3^*X*	0.1
Midazolam	0.2–200	0.9988	1/*X*^2^	*Y* = −5.23 × 10^−6^ + 1.91 × 10^−3^*X*	0.2

**Table 3:** The intra- and interday precision and accuracy of dexmedetomidine, dezocine, and midazolam in rat plasma (mean ± SD, 3 days, 6 replicates per day).

Analytes	Spiked conc. (ng/mL)	Intraday (*n*=6)			Interday (*n*=18)		
		Measured conc. (ng/mL)	Precision (RSD, %)	Accuracy (%)	Measured conc. (ng/mL)	Precision (RSD, %)	Accuracy (%)
Dexmedetomidine	0.1	0.119 ± 0.017	14.28	119.00	0.115 ± 0.015	13.04	115.00
	0.2	0.203 ± 0.022	10.83	101.50	0.197 ± 0.021	10.66	98.50
	5.0	4.63 ± 0.57	12.31	92.60	4.85 ± 0.39	8.04	97.00
	80	78.41 ± 4.95	6.31	98.01	79.36 ± 7.23	9.11	99.20

Dezocine	0.1	0.117 ± 0.015	12.82	117.00	0.114 ± 0.012	10.53	114.00
	0.2	0.206 ± 0.018	8.74	103.00	0.203 ± 0.020	9.85	101.50
	5.0	5.38 ± 0.51	9.48	107.60	5.26 ± 0.42	7.98	105.20
	80	85.15 ± 7.38	8.67	106.43	81.33 ± 5.86	7.21	101.26

Midazolam	0.2	0.226 ± 0.032	14.16	113.00	0.214 ± 0.028	13.08	107.00
	0.4	0.442 ± 0.052	11.76	110.50	0.414 ± 0.038	9.18	103.50
	10.0	10.78 ± 0.714	6.62	107.80	9.88 ± 0.82	8.30	98.80
	160	155.84 ± 13.86	8.89	97.40	159.14 ± 9.42	5.92	99.46

**Table 4:** Extraction recovery and matrix effect of dexmedetomidine, dezocine, and midazolam in rat plasma (mean ± SD, *n*=3).

Analytes	Spiked conc. (ng/mL)	Extraction recovery (%)		Matrix effect (%)	
		Mean ± SD	RSD	Mean ± SD	RSD
Dexmedetomidine	0.2	85.22 ± 8.72	10.23	90.76 ± 7.65	8.43
	5.0	88.98 ± 5.16	5.80	92.39 ± 3.27	3.54
	80	90.27 ± 4.55	5.04	96.82 ± 3.74	3.86

Dezocine	0.2	88.08 ± 7.44	8.45	91.47 ± 8.29	9.06
	5.0	95.63 ± 3.26	3.41	97.52 ± 1.98	2.03
	80	96.48 ± 2.88	2.99	92.33 ± 4.46	4.83

Midazolam	0.4	87.58 ± 8.24	9.41	93.62 ± 3.68	3.93
	10.0	96.92 ± 5.07	5.23	95.59 ± 2.29	2.39
	160	97.33 ± 2.67	2.74	92.25 ± 4.28	4.64

**Table 5:** Stability data of dexmedetomidine, dezocine, and midazolam in rat plasma under various storage conditions (mean ± SD, *n*=6).

Analytes	Spiked conc. (ng/mL)	Storage conditions (ng/mL)			
		Ambient temperature for 6 h	−20°C for 10 d	Three freeze-thaw cycles	Autosampler at 4°C for 24 h
Dexmedetomidine	0.2	0.206 ± 0.018	0.179 ± 0.021	0.212 ± 0.016	0.196 ± 0.011
	80	78.88 ± 9.25	76.29 ± 8.33	84.74 ± 7.65	81.16 ± 7.23

Dezocine	0.2	0.205 ± 0.017	0.214 ± 0.022	0.187 ± 0.026	0.193 ± 0.018
	80	77.45 ± 6.50	79.06 ± 9.19	74.82 ± 7.27	84.64 ± 5.83

Midazolam	0.4	0.380 ± 0.028	0.390 ± 0.040	0.434 ± 0.044	0.376 ± 0.024
	160	167.58 ± 19.14	145.22 ± 15.66	165.90 ± 8.76	172.46 ± 19.34

**Table 6:** Pharmacokinetic (PK) parameters of dexmedetomidine (20 *μ*g/kg), dezocine (80 *μ*g/kg), and midazolam (400 *μ*g/kg) following single intravenous administration in SD rats (mean ± SD, *n*=6).

PK parameters	Unit	Dexmedetomidine	Dezocine	Midazolam
AUC_(0–8 h)_^*∗*^	ng/mL·min	1336.05 ± 298.0	2317.92 ± 442.3	26,330.36 ± 6085.2
MRT_(0–8 h)_	min	131.49 ± 10.4	171.60 ± 13.6	89.42 ± 2.8
T_1/2z_	min	141.25 ± 61.4	225.39 ± 70.2	80.51 ± 19.3
C_2 min_	ng/mL	23.24 ± 5.0	22.37 ± 6.6	645.68 ± 157.5
*V*_d_	L/kg	2.68 ± 0.6	8.75 ± 2.2	1.778 ± 0.4
*C*_Lz/F_	L/min/kg	0.014 ± 0.004	0.028 ± 0.007	0.016 ± 0.004

^*∗*^AUC, area under the curve; MRT, mean residence time; *T*_1/2z_, half-life; *C*_2 min_, the concentration at 2 min; *V*_d_, apparent volume of distribution; *C*_Lz/F_, clearance.
